# COVID‐19 pandemic's relationship with enrollment at US Alzheimer's Disease Research Centers

**DOI:** 10.1002/alz.13706

**Published:** 2024-02-01

**Authors:** C. Elizabeth Shaaban, Hsing‐Hua Sylvia Lin, Melita Terry, Dianxu Ren, Jennifer H. Lingler

**Affiliations:** ^1^ Department of Epidemiology School of Public Health University of Pittsburgh Pittsburgh Pennsylvania USA; ^2^ Alzheimer's Disease Research Center University of Pittsburgh Pittsburgh Pennsylvania USA; ^3^ Department of Anesthesiology & Perioperative Medicine University of Pittsburgh School of Medicine Pittsburgh Pennsylvania USA; ^4^ Department of Neurology, School of Medicine University of Pittsburgh Pittsburgh Pennsylvania USA; ^5^ Department of Health & Community Systems, School of Nursing University of Pittsburgh Pittsburgh Pennsylvania USA

**Keywords:** brain health equity, dementia, recruitment and representation science, study participation

## Abstract

**INTRODUCTION:**

We aimed to characterize the COVID‐19 pandemic's relationship with enrollment in US Alzheimer's Disease Research Centers (ADRCs).

**METHODS:**

Using data on 10,105 participants from 30 ADRCs, we conducted interrupted time series analyses to assess the relationship of the pandemic with enrollment and calculate projected dates of enrollment recovery.

**RESULTS:**

Participants enrolled during the pandemic (vs pre‐pandemic) were more likely to have dementia and be referred by health professionals. The pandemic was associated with a 77% drop in enrollment, with projected trend recovery in March 2024 and 100% recovery in September 2024. COVID was associated with a 91% drop in Black/African American participants, compared to 71% in White participants. Enrollment of both Hispanic and female participants was declining 1.4% and 0.3%/month pre‐pandemic.

**DISCUSSION:**

Funders and researchers should account for ongoing COVID‐19 impact on ADRD research enrollment. Strategies to speed enrollment recovery are needed, especially for Black/African American and Hispanic groups.

**Highlights:**

- Tested COVID pandemic association with enrollment at Alzheimer's Disease Research Centers.
- During versus pre‐pandemic enrollees differed on demographic and clinical variables.
- Interrupted time series analyses: immediate 77% drop in enrollment related to COVID.
- Recovery projections: trend recovery in March 2024, 100% recovery in September 2024.
- Enrollment of African American and Hispanic participants should be prioritized.

## 1 BACKGROUND

The COVID‐19 pandemic's impact has been disproportionately felt based on self‐identified race, ethnicity, and gender as well as sex. As compared to non‐Hispanic White Americans, people from historically underserved racial and ethnic minority populations had greater COVID positivity and more severe disease,^1^ were hospitalized in greater proportions than they represent in state populations,^2^ and had lower vaccine coverage.^3^ Sex‐based COVID differences have been heterogenous, and some are due to social and contextual factors that can be gender‐related.^4^ For example, women are more likely than men to work as paid or unpaid caregivers as well as in other service industry positions leading to greater occupational exposure to COVID.^5^ Women have also been more likely than men to have permanent pandemic‐related job loss.^5^

Long preceding the pandemic, notable dementia‐related health disparities have existed.^6^ These disparities are pervasive and include differences in the prevalence, timely diagnosis, and clinical management of dementia, with individuals from historically underserved communities experiencing the most negative outcomes across each of these domains.^7^ For example, in an integrated health system‐based study, US dementia incidence varied approximately 60% across racial and ethnic groups, with people in the African American group having the highest risk and those in the Latino and White groups having intermediate risk.^8^ Global dementia prevalence by sex is greater for female older adults than male older adults in all age bands from 65 up.^9^ Because individuals from these sociodemographic groups bear a disproportionate burden of disease, they are arguably most in need of the benefits that can be conferred from participation in Alzheimer's disease and related dementias (ADRD) research. As such, it is imperative to ensure adequate representation of these individuals in clinical research on ADRD.^10^

**RESEARCH IN CONTEXT**

1. **Systematic review**: The authors searched PubMed for available reports on the impact of the COVID‐19 pandemic on enrollment into large‐scale, Alzheimer's disease research networks. While no such reports were identified, other research groups documented drops in clinical research enrollment for various health conditions ranging from 19% to 67% during the pandemic.
2. **Interpretation**: Our analysis of data from 30 National Institute on Aging‐funded Alzheimer's Disease Research Centers (ADRCs) quantified the association of the pandemic with enrollment within the Centers’ network by calculating an immediate 77% drop in enrollment. Findings make a significant and timely contribution to the field, estimating a during‐pandemic increase in enrollment of 1.5%/month, and projected recovery dates of March 2024 (trend recovery) and September 2024 (100% recovery).
3. **Future directions**: Innovative recruitment strategies will be required to ensure that the post‐pandemic ADRC participant pool is enriched for prodromal AD cases and is equitable in representation of Black/African American and Hispanic participants and women.

While fundamentally a matter of ensuring equitable access to the most promising treatments of the future, representation of diverse populations in ADRD research has additional implications. First, it is also a matter of scientific accuracy.^11^, ^12^, ^13^, ^14^ If we want to know which groups are at greatest risk of ADRD, who benefits or is harmed most by certain interventions, and address other questions, including those critical to reducing ADRD health disparities, research studies must include participants from a wide range of racial, ethnic, and sociodemographic backgrounds. Second, representation is an important avenue by which to enhance trust in clinical research and its findings.^13^ Recent research suggests that people from groups typically underrepresented in clinical research are more trusting that a drug treatment will benefit them when the clinical trials for drug approval were more representative.^15^ Through this lens, representation in research by people from a diverse range of self‐identified races, ethnicities, gender identities, and other groups is crucial to advancing health equity. Achieving such representation in ADRD clinical research requires intentionally inclusive research practices.

Prior to the pandemic, reports of severe underrepresentation of persons from racially and ethnically diverse backgrounds in ADRD research spurred multiple calls for inclusive recruitment approaches.^14^, ^16^, ^17^ The United States’ National Institute on Aging (NIA)‐funded Alzheimer's Disease Research Centers (ADRCs) network is an important part of ADRD research and a recruitment source for many cutting‐edge clinical trials. Because ADRCs tend to be highly selected samples not representative of the general US population, these centers were the focus of numerous initiatives to promote diversity and inclusion in the years leading up to the pandemic.^18^, ^19^, ^20^ In the context of the pandemic, substantial losses of participation among people from historically underserved racial and ethnic groups or women would undermine progress on the aims of reducing ADRD health disparities and promoting brain health equity and justice. Thus, our objective was to characterize the relationship of the COVID‐19 pandemic with enrollment in the NIA‐funded ADRCs overall and by race, ethnicity, and sex.

## 2 METHODS

### 2.1 Study data

We carried out a secondary data analysis evaluating National Alzheimer Coordinating Center (NACC) baseline study visit data from ADRCs that had ≥5 years of data and study visits after 2020. Data from the September 2022 NACC data freeze were used in this analysis. Baseline study visits took place from January 2017 through June 2022, providing approximately three years of stable pre‐pandemic data and slightly more than two years of pandemic data on enrollment. Deidentified NACC data are publicly available from https://naccdata.org/ with a data request.

### 2.2 Human subjects

This study was reviewed and approved by each site's Institutional Review Board (IRB), and all study participants underwent the informed consent process before completing study assessments.

### 2.3 Monthly enrollment rate

The mean monthly pre‐pandemic enrollment for all included ADRCs from years 2017 to 2019 was *N* = 205. We calculated the monthly enrollment rate by normalizing each month's enrollment by the mean monthly pre‐pandemic enrollment.

### 2.4 COVID‐19 pandemic date

The World Health Organization (WHO) declared the COVID‐19 outbreak a pandemic on March 11, 2020.^21^ We used this date to categorize NACC baseline visits pre‐pandemic versus during the pandemic.

### 2.5 Characteristics and clinical variables

Note that labeling of participants’ sociodemographic characteristics in the Methods and Results sections reflects variable labeling in the NACC Uniform Data Set (UDS).^22^ Participants’ characteristics include sex (male or female; the NACC UDS does not presently collect gender), age, self‐reported race (White, Black or African American, American Indian or Alaska Native, Native Hawaiian or Pacific Islander, Asian, Multiracial [reports more than one race], unknown or ambiguous), self‐reported Hispanic/Latino ethnicity (yes or no), years of education, current marital status (married, widowed, divorced, separated, never married [or marriage was annulled], living as married/domestic partner, or other or unknown), and living situation (lives alone, lives with spouse or partner, lives with relative or friend, lives with group, other, or unknown). Clinical variables included level of independence (able to live independently, requires some assistance with complex activities, requires some assistance with basic activities, completely dependent, or unknown), cognitive status (normal cognition; cognitively impaired, not mild cognitive impairment [MCI]; MCI; or dementia), Montreal Cognitive Assessment (MoCA) score, the primary reason for coming to the ADRC (to participate in a research study, to have a clinical evaluation, both [to participate in a research study and to have a clinical evaluation], or unknown), and the principal referral source (non‐professional contact: self/relative/friend; professional contact: clinician, nurse, doctor, or others; other; or unknown). Co‐participant characteristics included sex, Hispanic ethnicity, race, relationship to the participant (not available; spouse, partner, or companion; child; sibling; other relative; friend, neighbor, co‐worker; paid caregiver, health care provider, or clinician), years that the co‐participant has known the participant, and living status with the participant (lives with the participant, yes or no).

### 2.6 Statistical analysis

Kruskal‐Wallis or Pearson's chi‐square tests were used to test whether distributions of continuous and categorical variables differed between those enrolled pre‐pandemic and those enrolled during the pandemic. We compared the pre‐pandemic and during pandemic enrollment rates using interrupted time series analysis (ITSA) by segmented linear regression.^23^ ITSA estimated the changes in level (ie, immediate change in enrollment rate) at the beginning of the COVID‐19 pandemic and the trends (increase or decrease of the enrollment rate per month) pre‐pandemic and during the pandemic. We report coefficients, 95% confidence intervals (CIs), and *p*‐values.

**FIGURE 1:**
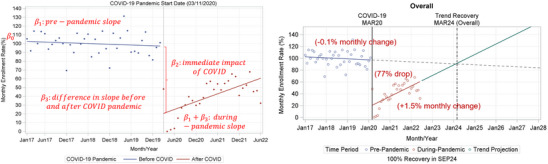
Interrupted time series analysis of the impact of the COVID‐19 pandemic on enrollment and the projected pre‐pandemic enrollment recovery date, in the full study sample.

We followed the standard ITSA segmented regression model:

$$Y_{t}=\beta_{0}\;+\beta_{1}\times time_{t}+\beta_{2}\times COVID_{t}+\beta_{3}\times time\;since\;COVID+\varepsilon_{t\;},$$

where *Y_t_* is the monthly enrollment rate at timepoint *t*, *time_t_* is the time in months since the start of the study from the 1st month of the pre‐pandemic study period (ie, January 2017), *COVID_t_* represents the pandemic as a binary variable with pre‐pandemic months designated as 0 and during pandemic months designated as 1. Time since COVID is 0 for the pre‐pandemic period and the first month of COVID (March 2020), 1 for April 2020, 2 for May 2020, and so on. *β*_0_ is the intercept and represents the initial outcome *Y* (eg, monthly enrollment rate) at the 1st month of the study period, while *β*_1_ is the pre‐pandemic period slope (eg, change in enrollment rate per month), and *β*_3_ is the difference in slopes between pre‐ and during‐COVID periods. The slope during the COVID pandemic is the sum of *β*_1_ and *β*_3_. *β*_2_ is the level change in enrollment rate, defined as the immediate change associated with COVID‐19 (Figure 1, left).

We checked the overall autocorrelation of data across two periods through the autocorrelation functions plot and the Durban‐Watson statistics. If overall autocorrelation across the pre‐ and during‐COVID periods was identified, the regression standard errors were adjusted using the Newey‐West method.^24^

Based on the estimates from the ITSA, we calculated projected recovery dates for the overall study sample and stratified by race, ethnicity, and sex. Evaluation of enrollment by race includes only Black/African American and White participants due to small numbers in other racial groups. The pre‐pandemic trendline was projected on the assumption that, had the COVID‐19 pandemic not occurred, we would have maintained the same enrollment trajectory we were already following, without accounting for any other interventions that took place after the pandemic. Two recovery dates were projected. The trend recovery date was determined as the point where the during‐pandemic trendline intersected with the pre‐pandemic trendline, allowing us to estimate the potential recovery date by assuming that the pre‐pandemic trend would continue as it was. The 100% recovery date was projected based on when the during‐pandemic trend returned to 100% of the pre‐pandemic average monthly enrollment.

Statistical analyses were carried out in SAS version 9.4.^25^

## 3 RESULTS

### 3.1 Participant characteristics

We included data on 10,105 participants (mean age, 69.4 years; 58% female; 10% Hispanic/Latino; 14% Black/African American) from 30 ADRCs.

**FIGURE 2:**
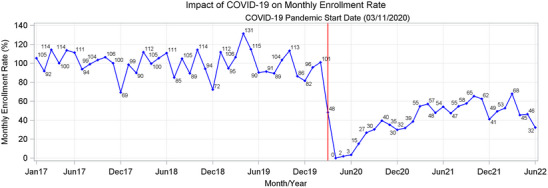
Monthly Alzheimer Disease Research Center enrollment rate from January 2017 to June 2022.

Enrollment dropped to 0% of pre‐pandemic levels in April 2020; enrollment recovered to a maximum of only 68% of pre‐pandemic levels through June 2022 (Figure 2). Participants enrolled during the pandemic (vs pre‐pandemic) were less likely to be women, White, Black/African American, cognitively normal, able to live independently, and to actually live alone (Table 1). They were slightly younger; slightly more educated; and more likely to be Hispanic/Latino, be married, be referred by a doctor, nurse, or other professional; and come in for a clinical or mixed clinical and research study evaluation (all *p* < 0.05; Table 1). Compared to pre‐pandemic co‐participants, during‐pandemic co‐participants were more likely to be female, spouses or partners, and to reside with the participant. They were less likely to be Black/African American or multiracial (all *p* < 0.05; Table 1).

**TABLE 1:** Baseline characteristics by COVID pandemic start date (March 11, 2020).

Variable	All *n* = 10,105	Pre‐pandemic *n* = 7853	During‐pandemic *n* = 2252	*p*‐value
Female sex	5838 (58%)	4622 (59%)	1216 (54%)	**<0.001**
Age, years	69.4 (9.8)	69.6 (9.9)	68.6 (9.4)	**<0.001**
Hispanic/Latino ethnicity	976 (10%)	729 (9%)	247 (11%)	**0.005**
Race				**0.013**
White	7750 (77%)	6048 (77%)	1702 (76%)	
Black or African American	1391 (14%)	1097 (14%)	294 (13%)	
American Indian or Alaska Native	65 (1%)	45 (1%)	20 (1%)	
Native Hawaiian or Pacific Islander	11 (0%)	8 (0%)	3 (0%)	
Asian	372 (4%)	271 (3%)	101 (4%)	
Multiracial	292 (3%)	226 (3%)	66 (3%)	
Unknown or ambiguous	224 (2%)	158 (2%)	66 (3%)	
Education, years^a^	15.8 (3.1)	15.8 (3.1)	16.0 (2.9)	**0.001**
Marital status				**<0.001**
Married	6759 (67%)	5184 (66%)	1575 (70%)	
Widowed	1080 (11%)	891 (11%)	189 (8%)	
Divorced	1305 (13%)	1044 (13%)	261 (12%)	
Separated	125 (1%)	97 (1%)	28 (1%)	
Never married (or marriage was annulled)	584 (6%)	448 (6%)	136 (6%)	
Living as married/domestic partner	184 (2%)	140 (2%)	44 (2%)	
Other or unknown	68 (1%)	49 (1%)	19 (1%)	
Living situation				**0.043**
Lives alone	2124 (21%)	1697 (22%)	427 (19%)	
Lives with spouse or partner	6365 (63%)	4891 (62%)	1474 (65%)	
Lives with relative or friend	644 (6%)	494 (6%)	150 (7%)	
Lives with group	876 (9%)	691 (9%)	185 (8%)	
Other	28 (0%)	23 (0%)	5 (0%)	
Unknown	68 (1%)	57 (1%)	11 (0%)	
Level of independence				**<0.001**
Able to live independently	7500 (74%)	5870 (75%)	1630 (72%)	
Requires some assistance with complex activities	1712 (17%)	1265 (16%)	447 (20%)	
Requires some assistance with basic activities	548 (5%)	423 (5%)	125 (6%)	
Completely dependent	178 (2%)	144 (2%)	34 (2%)	
Unknown	167 (2%)	151 (2%)	16 (1%)	
Cognitive status at UDS visit				**<0.001**
Normal cognition	4556 (45%)	3587 (46%)	969 (43%)	
Cognitively impaired, not MCI	400 (4%)	343 (4%)	57 (3%)	
MCI	2507 (25%)	1950 (25%)	557 (25%)	
Dementia	2642 (26%)	1973 (25%)	669 (30%)	
MoCA Total Score, uncorrected^b^	22.3 (6.1)	22.4 (6.0)	22.0 (6.3)	**0.016**
MoCA Total Score, corrected for education^c^	22.5 (6.0)	22.6 (5.9)	22.1 (6.2)	**0.008**
Primary reason for coming to ADRC				**<0.001**
Research study	7071 (70%)	5656 (72%)	1415 (63%)	
Clinical evaluation	522 (5%)	388 (5%)	134 (6%)	
Research study and a clinical evaluation	2481 (25%)	1788 (23%)	693 (31%)	
Unknown	31 (0%)	21 (0%)	10 (0%)	
Principal referral source				**<0.001**
Non‐professional contact: self/relative/friend	3740 (37%)	3045 (39%)	695 (31%)	
Professional contact: clinician, nurse, doctor, or others	5378 (53%)	4062 (52%)	1316 (58%)	
Other	729 (7%)	535 (7%)	194 (9%)	
Unknown	258 (3%)	211 (3%)	47 (2%)	
Co‐participant female sex	6273 (62%)	4843 (62%)	1430 (63%)	**0.036**
Co‐participant Hispanic/Latino ethnicity	909 (9%)	716 (9%)	193 (9%)	0.10
Co‐participant race				**0.032**
Not available	231 (2%)	194 (2%)	37 (2%)	
White	7521 (74%)	5830 (74%)	1691 (75%)	
Black or African American	1305 (13%)	1037 (13%)	268 (12%)	
American Indian or Alaska Native	41 (0%)	34 (0%)	7 (0%)	
Native Hawaiian or Pacific Islander	12 (0%)	9 (0%)	3 (0%)	
Asian	381 (4%)	279 (4%)	102 (5%)	
Multiracial	257 (3%)	204 (3%)	53 (2%)	
Unknown or ambiguous	357 (4%)	266 (3%)	91 (4%)	
Co‐participant relationship to participant				**0.014**
Not available	231 (2%)	194 (2%)	37 (2%)	
Spouse, partner, or companion	6090 (60%)	4672 (59%)	1418 (63%)	
Child	1787 (18%)	1388 (18%)	399 (18%)	
Sibling	545 (5%)	429 (5%)	116 (5%)	
Other relative	291 (3%)	235 (3%)	56 (2%)	
Friend, neighbor, co‐worker	1117 (11%)	900 (11%)	217 (10%)	
Paid caregiver, health care provider, or clinician	44 (0%)	35 (0%)	9 (0%)	
How long has the co‐participant known the participant^d^	39.7 (17.1)	39.9 (17.3)	39.2 (16.2)	0.06
Co‐participant lives with the participant	6531 (65%)	5024 (64%)	1507 (67%)	**0.007**

*Note*: Bold *p*‐values are significant at *ɑ* = 0.05.

Abbreviations: UDS, Uniform data set; MCI, mild cognitive impairment; MoCA, Montreal Cognitive Assessment; ADRC, Alzheimer's Disease Research Center.

^a^ Total *N* = 10,018, Pre‐pandemic *N* = 7775, During‐pandemic *N* = 2243.

^b^ Total *N* = 9243, Pre‐pandemic *N* = 7394, During‐pandemic *N* = 1849.

^c^ Total *N* = 9184, Pre‐pandemic *N* = 7337, During‐pandemic *N* = 1847.

^d^ Total *N* = 9507, Pre‐pandemic *N* = 7370, During‐pandemic *N* = 2137.

### 3.2 Enrollment patterns and projected recovery dates

The Durban‐Watson statistic was 1.89, indicating autocorrelation was detected in the sample, and it was driven by monthly patterns in enrollment (Figure 2). Thus, the regression standard errors were adjusted using the Newey‐West method with a lag of 12 months. ITSA demonstrated that the pandemic was associated with an immediate 77% drop in enrollment rate, with an estimated post‐pandemic increase in enrollment of 1.5%/month, leading to projected overall recovery dates of March 2024 for trend recovery and September 2024 for 100% recovery (Table 2; Figure 1, right).

**TABLE 2:** Interrupted time series analyses for the full study sample overall.

Parameter	Estimate	95% CI	*p*‐value	Interpretation
β_0_	102.50	(98.86, 106.15)	<0.001	The starting (intercept) enrollment rate is 102.50%
β_1_	−0.14	(−0.32, 0.04)	0.12	The change of enrollment rate is −0.14% per month, but it is only borderline significantly different from zero.
β_2_	−76.56	(−87.77, −65.36)	<0.001	The immediate impact of COVID is a 76.56% drop in monthly enrollment rate.
β_3_	1.62	(0.77, 2.47)	<0.001	The slopes before and after COVID are significantly different by 1.62%.
β_1_ + β_3_	1.48	(0.68, 2.28)	<0.001	The change of enrollment rate is 1.48% per month during the pandemic.

*Note*: The model accounts for a lag = 12 autocorrelation.

**FIGURE 3:**
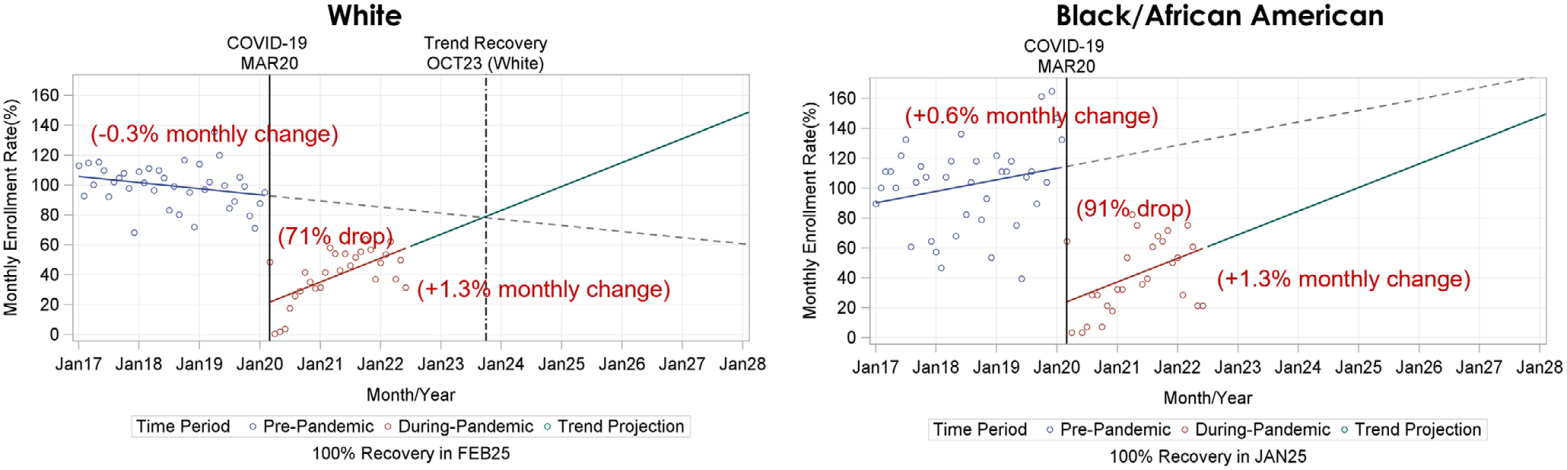
Interrupted time series analysis of the impact of the COVID‐19 pandemic on enrollment and the projected pre‐pandemic enrollment recovery date, stratified by race.

Stratified ITSA analyses showed the relationship of COVID‐19 with enrollment rate and recovery dates differed by race, ethnicity, and sex. COVID‐19 was associated with a 91% drop in enrollment for Black/African American participants, compared to a 71% drop for White participants (Table 3, Figure 3). Black/African American participant enrollment was increasing non‐significantly by about 0.6% per month versus White participant enrollment which was decreasing 0.3% per month pre‐pandemic (Table 3, Figure 3), likely contributing to patterns with trend recovery. During the pandemic, the enrollment rate trends were the same (+1.3% per month) for Black/African American and White participants (Table 3, Figure 3). Projected trend recovery varied by race; we projected October 2023 for White participants, but we could not predict a recovery date for Black/African American participants by January 2028 (Figure 3). White participants’ enrollment was projected to hit 100% of pre‐pandemic levels by February 2025, with that for Black/African American participants reaching 100% by January 2025 (Figure 3).

**TABLE 3:** Interrupted time series analyses stratified by race, ethnicity, and sex.

Parameter	Estimate	95% CI	*p*‐value	Estimate	95% CI	*p*‐value
	**White participants**			**Black participants**		
β_0_	105.88	(101.99 to 109.76)	<0.001	90.21	(72.04 to 108.38)	<0.001
β_1_	−0.34	(−0.61 to −0.07)	0.015	0.64	(−0.31 to 1.60)	0.192
β_2_	−71.15	(−82.14 to −60.16)	<0.001	−90.59	(−121.68 to −59.50)	<0.001
β_3_	1.68	(0.78 to 2.57)	0.001	0.68	(−0.67 to 2.02)	0.329
β_1_ + β_3_	1.33	(0.56 to 2.11)	0.001	1.32	(0.19 to 2.45)	0.022
	**Non‐Hispanic/Latino**			**Hispanic/Latino**		
β_0_	100.31	(96.00 to 104.61)	<0.001	124.81	(113.57 to 136.05)	<0.001
β_1_	−0.01	(−0.21 to 0.18)	0.886	−1.40	(−1.89 to −0.92)	<0.001
β_2_	−77.32	(−89.84 to −64.80)	<0.001	−62.59	(−74.35 to −50.83)	<0.001
β_3_	1.33	(0.39 to 2.27)	0.007	4.42	(3.73 to 5.12)	<0.001
β_1_ + β_3_	1.32	(0.43 to 2.20)	0.004	3.02	(2.51 to 3.52)	<0.001
	**Male participants**			**Female participants**		
β_0_	99.25	(95.03 to 103.47)	<0.001	104.76	(98.95 to 110.56)	<0.001
β_1_	0.05	(−0.13 to 0.23)	0.584	−0.27	(−0.53 to −0.01)	0.042
β_2_	−75.25	(−89.13 to −61.37)	<0.001	−77.26	(−87.45 to −67.08)	<0.001
β_3_	1.42	(0.39 to 2.45)	0.009	1.75	(0.99 to 2.51)	<0.001
β_1_ + β_3_	1.47	(0.47 to 2.47)	0.004	1.48	(0.81 to 2.14)	<0.001

*Note*: The models account for a lag = 12 autocorrelation.

**FIGURE 4:**
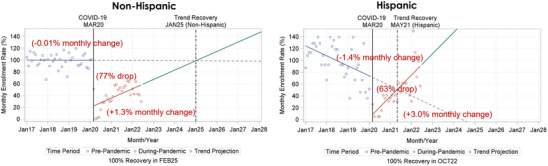
Interrupted time series analysis of the impact of the COVID‐19 pandemic on enrollment and the projected pre‐pandemic enrollment recovery date, stratified by Hispanic ethnicity.

COVID‐19 was associated with a 63% drop in enrollment for Hispanic participants, compared to a 77% drop for non‐Hispanic participants. Hispanic participant enrollment was declining nearly 1.4% per month pre‐pandemic versus a stable enrollment rate in non‐Hispanic participants (−0.01%), which likely impacted trend recovery projections (Table 3, Figure 4). The enrollment rate was increasing by 3.0% per month during the pandemic among Hispanic versus 1.3% for non‐Hispanic participants (Table 3, Figure 4). Projected recovery varied by ethnicity. Hispanic participant enrollment recovered earlier than non‐Hispanic participant enrollment (trend recovery: May 2021 vs January 2025; 100% recovery: October 2022 vs February 2025; Figure 4).

**FIGURE 5:**
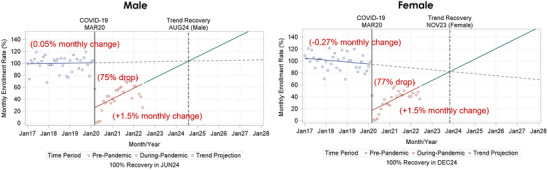
Interrupted time series analysis of the impact of the COVID‐19 pandemic on enrollment and the projected pre‐pandemic enrollment recovery date, stratified by sex.

COVID‐19 was associated with similar drops in enrollment for female and male participants (77% and 75%, respectively; Table 3, Figure 5). Female participant enrollment was declining by about 0.3% per month pre‐pandemic versus stable enrollment rate (+0.1%) in males, which likely impacted trend recovery projections (Table 3, Figure 5). The enrollment rate trend was similar during the pandemic (+1.5% per month) for male and female participants (Table 3, Figure 5). Female participant trend recovery was sooner than that for male participants (November 2023 vs August 2024; Figure 5). One hundred percent recovery was estimated in December 2024 for female participants and June 2024 for male participants (Figure 5).

## 4 DISCUSSION

European and North American research groups have documented drops in clinical research enrollment for various health conditions ranging from 19% to 67% during the pandemic.^26^, ^27^, ^28^ The current report is the first to quantify this phenomenon in the context of ADRD research and identify projected recovery dates to pre‐pandemic enrollment levels. Our analysis demonstrated that the COVID‐19 pandemic was associated with an immediate 77% drop in the enrollment rate at ADRCs. Projected recovery dates were March of 2024 for trend recovery and September 2024 for 100% recovery, while some subgroups are estimated to take into 2028 or beyond to recover. Because the ADRC network is a major source of recruitment into federally‐ and industry‐sponsored clinical trials (as well as other study types), these findings indicate that the potential pool of clinical trial candidates will continue to be constrained over the next 1 to 5 years depending on the subgroup. Next, we discuss key ways in which participants in this during‐pandemic pool may differ from those in the pre‐pandemic era, and outline implications for accelerating the enrollment of study participants.

### 4.1 Pre‐ and during‐pandemic differences

Participants enrolled during the pandemic were less likely to be cognitively normal and to live independently compared to those who enrolled pre‐pandemic, and this corresponds with our observations that such individuals were presenting at least in part for a clinical evaluation of symptoms and were more likely to be referred by a clinician. They were slightly younger, slightly more educated, and more likely to be men. To the extent that pandemic era enrollment in ADRCs reflects prioritization of the need for a research grade cognitive evaluation, these findings might be interpreted as indicating that cognitive decline is more likely to be problematized when occurring in men and those who are younger and more educated. These findings raise the possibility that the ADRCs may emerge from the pandemic with a more symptomatic pool of participants as compared to the pre‐pandemic pool. While this suggests that ADRCs may have fulfilled an important need for those seeking cognitive evaluations during the pandemic, efforts to enrich the pool for prodromal AD may be needed.

### 4.2 Enrollment declines

While decreased ADRC enrollment during the pandemic occurred across racial and ethnic groups, greater immediate declines were seen for Black/African American participants than White participants, and this may reflect a deprioritization of research participation in the face of disproportionate health risks and caregiving needs experienced by those in the Black/African American group. Greater immediate declines were seen for non‐Hispanic versus Hispanic participants. This pattern might be expected if the ADRCs were fulfilling an unmet need for clinical care among people identifying as Hispanic since this group was heavily impacted by the pandemic, and it is at least partially influenced by the decreasing monthly pre‐pandemic enrollment for those in the Hispanic group.

### 4.3 Projected recovery dates

Given that resuming pre‐pandemic efforts to increase representation of diverse participants is a shared objective for researchers across the ADRC network, we sought to identify two projected enrollment recovery dates for participants identifying as Black/African American and those identifying as Hispanic: recovery to the pre‐pandemic trend and recovery to 100% of the pre‐pandemic average monthly enrollment. In the case of Black/African American participants, a return to the pre‐pandemic trend, which had seen increasing monthly enrollment, or better, should be the goal. We could not predict this trend recovery date for Black/African American participants, indicating that the during‐pandemic enrollment rate of Black/African American participants is not sufficiently rapid to catch up to the pre‐pandemic trend level of increasing monthly enrollment in the foreseeable future. Projected recovery also varied by ethnicity, with Hispanic participant enrollment recovering earlier than non‐Hispanic participant enrollment, but this was largely because Hispanic participant enrollment was declining nearly 1.4%/month pre‐pandemic. This adds up to worrisomely large enrollment declines over the course of a year. Thus, in the case of Hispanic participants, a return to 100% pre‐pandemic average or more should be the recovery goal rather than the pre‐pandemic trend. Overall, participants in the NACC database are overwhelmingly White and non‐Hispanic, with our total sample comprised of 77% White participants and 90% of non‐Hispanic ethnicities.

We found that recovery date projections also varied by sex, with female participant trend enrollment recovering sooner than male participant trend enrollment. Again, in this instance, this appeared to be a function of pre‐pandemic enrollment declining 0.3%/month for female participants, and when considering 100% recovery instead, female participants’ enrollment will recover 6 months later than that of male participants. Monthly enrollment gains during the pandemic did not differ by sex, but female participant enrollment should be accelerated to reach 100% recovery.

Our analysis was not designed to shed light on why Hispanic and female participants’ enrollment rates were declining pre‐pandemic, and so the cause(s) of these phenomena remain unclear. Pre‐pandemic secular trends may already have been resulting in deprioritization of gold standard cognitive research evaluations for these groups; additional research would be required to further clarify these patterns.

### 4.4 Recommendations to spur enrollment recovery

The ADRC network should develop strategies to speed enrollment recovery for women and especially for the Black/African American and Hispanic groups. Prior work suggests a number of factors could increase recruitment, including some specifically focused on groups underrepresented in research, such as community outreach, tailored messaging, giving back to the community, peer recruiters, reducing participant burden, and partnering with health care providers.^14^, ^29^, ^30^, ^31^ Specific approaches likely need to be tailored to the group and context, although this creates a tension between effective recruitment and creating differences between groups due to different recruitment sources and strategies. For example, it has previously been shown within the ADRCs that referral source is likelier to be non‐clinical for Black/African American participants and clinical for non‐Hispanic White participants, and that these differences bias statistical models of relationships being tested.^32^

Few of these recruitment approaches have been directly tested via intervention trials. However, among those that have been, work from randomized controlled trials suggests that paying participants an incentive for participation increases both response and consent rates.^33^, ^34^ Furthermore, data from the smoking cessation trial of Halpern and colleagues’ RETAIN study^34^ shows that participant payments increased participation more among Black/African American participants than among White participants, suggesting that incentives may enhance the diversity of study enrollment (S. Halpern, personal communication, 4/19/2023), although another recent non‐interventional survey‐based study on recruitment into a hypothetical longitudinal AD cohort study did not find differential increases to minority enrollment.^35^ While we are extrapolating from the randomized controlled trial context to the observational ADRC context, given that many centers in the network do not offer participant incentives, this may be an impactful, evidence‐based intervention to speed enrollment recovery rates. Best practices regarding ADRC participant payment are newly available via the NACC website (https://files.alz.washington.edu/documentation/remuneration‐guidelines.pdf).

The figures on ADRC enrollment by race and ethnicity underscore the appropriateness of NIA's specifications, in the new ADRC request for applications document, that centers enhance enrollment of groups locally historically underrepresented in ADRD research, especially those at high risk, and provide adequate staffing to support community engagement and participant retention activities.^36^ Furthermore, new and exploratory ADRCs designed to enroll specific populations such as Hispanic participants are likely critical to the network's enrollment recovery over the next several years.

### 4.5 Limitations

Several important caveats should be kept in mind when interpreting our study results. First, this data is based on 30 ADRCs, but NIA currently funds 33 ADRCs around the United States as well as four exploratory ADRCs designed to include more diverse populations.^37^ Because we needed to compare pre‐ and during‐pandemic enrollment, we could only include ADRCs that had enrollment data pre‐2020 as well as during‐pandemic enrollment data. Thus, new, or previously funded centers could not be included in these analyses, but may contribute to enrollment of specific subgroups.

Several statistical considerations are important for interpreting the results. First, several requirements for a causal interpretation of this ITSA analysis were not met, and thus our findings do not rise to the level of demonstrating causality. A causal interpretation requires a realistic control group without exposure to COVID‐19, which was not feasible here. Furthermore, these models and projections are based on data through June 2022; ITSA results and projected recovery dates would likely vary with inclusion of more data. In addition, the pre‐pandemic trendline was projected without accounting for any other confounders or interventions taking place after the pandemic. However, numerous other potential competing interventions such as other policies and norms have changed across the pandemic. For example, mental health care needs and service delivery changed over the pandemic.^38^ We did not address such potential competing interventions here. Second, because our primary analysis was the overall association of COVID‐19 with enrollment and our subgroup analyses were exploratory, we did not correct for multiple comparisons. Third, the sample sizes were imbalanced across some subgroups. This may lead to differing confidence intervals across subgroups but would not be expected to change the effect sizes we report.

### 4.6 Strengths

The key strengths of this work are the large sample size allowing for greater precision with estimates and subgroup analyses to better understand race, ethnicity, and sex‐specific enrollment patterns relating to the COVID pandemic. ITSA analysis can quantify the immediate COVID‐19‐associated change in the enrollment rate as well as the change in monthly enrollment rate pre‐pandemic versus during pandemic. Based on ITSA estimates, we were able to project trend recovery dates and 100% recovery dates for the overall study sample and by subgroups to inform ongoing research projects using ADRC data. In addition, this work addresses an important and timely problem for which we can develop innovative yet practical solutions.

### 4.7 Conclusions

Funders and researchers should acknowledge and account for ongoing COVID‐19 impact on ADRD research enrollment. Innovative recruitment strategies will be required to ensure that the post‐pandemic ADRC participant pool is enriched for prodromal AD cases and is equitable in the representation of women, and particularly to speed enrollment of people identifying as Black/African American and Hispanic.

## CONFLICT OF INTEREST STATEMENT

C. Elizabeth Shaaban, Jennifer H. Lingler, and Melita Terry disclose that they are faculty or staff funded by the University of Pittsburgh ADRC ORE Core. C. Elizabeth Shaaban is chair of the ISTAART PIA to Elevate Early Career Researchers (PEERs) and co‐chair of the Sex and Gender Special Interest Group of the Diversity and Disparities PIA in ISTAART. Jennifer H. Lingler is past chair of the Steering Committee for NIA‐funded ADRC ORE Cores and has consulted for Genentech and Biogen. Dianxu Ren and Hsing‐Hua Sylvia Lin have nothing to disclose. Author disclosures are available in the supporting information.

## CONSENT STATEMENT

This study was reviewed and approved by each site's Institutional Review Board (IRB), and all study participants underwent the informed consent process before completing study assessments.

## Supporting information

Supporting Information
